# Meetings of Societies

**Published:** 1930

**Authors:** 


### Meetings of Societies.

### Bristol Medico-Chirurgical Society.

SESSION 1930-31.

The Annual General Meeting was held in the Physiological Lecture Theatre of the University on Wednesday, 8th October, 1930, at 8.15 p.m.

Mr. C. F. Walters was in the Chair, seventy-eight members being present.

The minutes of the previous meeting were read and confirmed.

The President then referred to the deaths of Colonel J. Paul Bush and Colonel T. M. Carter, old members of the Society. The members present signified their respect for their past colleagues and regret for their loss by standing.

Two ladies and twelve gentlemen were proposed for election to the membership of the Society.

The President, Mr. C. F. Walters, then resigned the Chair to his successor, Professor D. C. Rayner.

Mr. T. Carwardine proposed a vote of thanks to Mr. Walters for his services during the past year ; this was seconded by Mr. Walker and carried with acclamation, and Mr. Walters replied.

Professor D. C. Rayner then gave his inaugural address on **" The Influence of the Endocrine Glands on Obstetrics and Gynæcology "** (to appear in our next issue).

Dr. Watson-Williams proposed a vote of thanks to the President for his address; this was seconded by Professor Hey Groves and carried with acclamation, and Professor Rayner replied.

The Hon. Secretary and Hon. Treasurer then gave their reports, and Mr. A. L. Flemming, at the request of the Editor, gave a short report on the *Journal.*

### BRISTOL MEDICO-CHIRURGICAL SOCIETY.

### INCOME AND EXPENDITURE ACCOUNT for the year ended 30th September, 1930.

### INCOME.

| | £ | s. | d. | £ | s. | d. |
|---|---|---|---|---|---|---|
| Members' Subscriptions .. | 263 | 9 | 0 | | | |
| Do., outstanding for 1929–1930 .. .. .. .. .. | 63 | 0 | 0 | | | |
| | | | | 326 | 9 | 0 |
| Interest on Bank Deposits .. .. | | | | 4 | 0 | 9 |
| Interest on £500 4 per cent. Consolidated Stock .. .. .. .. .. | | | | 15 | 15 | 0 |
| British Medical Association Refund of ½ share X-ray Viewing Screen | | | | 4 | 10 | 0 |
| | | | | £350 | 14 | 9 |

### EXPENDITURE.

| | £ | s. | d. | £ | s. | d. | £ | s. | d. |
|---|---|---|---|---|---|---|---|---|---|
| Grant to *The Bristol Medico-Chirurgical Journal* .. .. .. | | | | | | | 275 | 4 | 11 |
| Librarian—Honorarium .. .. .. | | | | | | | 15 | 0 | 0 |
| Miscellaneous Expenses— | | | | | | | | | |
| Goodway–Refreshments | | | | 2 | 5 | 0 | | | |
| Postages and Sundries | | | | 9 | 8 | 8 | | | |
| Printing and Stationery | | | | 15 | 16 | 6 | | | |
| Audit Fee .. .. .. | | | | 2 | 2 | 0 | | | |
| Lantern and Cinematograph at Meetings .. | | | | 4 | 4 | 0 | | | |
| University Marshal .. | | | | 2 | 0 | 0 | | | |
| Lewis's Medical and Scientific Library— | | | | | | | | | |
| Subscription | 4 | 4 | 0 | | | | | | |
| Postages .. | 0 | 6 | 5 | | | | | | |
| | | | | 4 | 10 | 5 | | | |
| | | | | | | | 40 | 6 | 7 |
| Balance, being excess of Income over Expenditure carried to Capital Account .. .. .. .. .. .. | | | | | | | 20 | 3 | 3 |
| | | | | | | | £350 | 14 | 9 |

### BALANCE SHEET as at 30th September, 1930.

### CAPITAL AND LIABILITIES.

| | £ | s. | d. | £ | s. | d. |
|---|---|---|---|---|---|---|
| Sundry Creditors .. .. | | | | 37 | 1 | 1 |
| Capital as at 30th Sept., 1929 .. .. .. .. .. | 620 | 14 | 8 | | | |
| *Add* Arrears of Subscriptions paid up .. .. | 15 | 11 | 0 | | | |
| | 636 | 5 | 8 | | | |
| Balance of Income and Expenditure Account as above .. .. .. .. .. | 20 | 3 | 3 | | | |
| | | | | 656 | 8 | 11 |
| | | | | £693 | 10 | 0 |

### ASSETS.

| | £ | s. | d. | £ | s. | d. |
|---|---|---|---|---|---|---|
| Cash at Bank— | | | | | | |
| Current Account .. .. | 39 | 10 | 9 | | | |
| Deposit Account .. .. | 153 | 10 | 3 | | | |
| | | | | 193 | 1 | 0 |
| £500 4 per cent. Consolidated Stock at cost .. .. .. .. .. .. .. .. .. | | | | 437 | 9 | 0 |
| Members' Subscriptions for 1929–1930 outstanding .. .. .. .. .. .. | | | | 63 | 0 | 0 |
| | | | | £693 | 10 | 0 |

Audited and found correct,

H. E. KEELER,

*Chartered Accountant,*

*Auditor.*

16–18 Clare Street, Bristol,

*6th October*, 1930.

Dr. J. A. Nixon, seconded by Mr. C. F. Walters, proposed a vote of thanks to the Hon. Secretary and Hon. Treasurer for their reports and their services.

Dr. J. R. Charles was elected to the office of President-Elect, on the proposition of Dr. J. A. Nixon, seconded by Dr. Wills.

Mr. A. L. Flemming proposed and Mr. C. F. Walters seconded the re-election of Mr. W. A. Jackman as Hon. Secretary. This was carried.

Mr. E. Watson Williams proposed the re-election of Mr. A. E. Iles as Hon. Treasurer, this was seconded by Dr. Logan and carried.

Dr. G. Parker was elected Hon. Medical Librarian on the proposal of Mr. A. E. Iles, seconded by Dr. Fells.

The following gentlemen were elected to serve on the Committee, proposed by Dr. Lacy Firth and seconded by Dr. Carey Coombs :—

Mr. Duncan Wood.

Mr. Chitty.

Dr. R. S. Statham

Dr W. K. Wills.

Dr. Kyle.

Mr. Hey Groves.

Dr. Kennedy.

Mr. C. F. Walters.

Mr. A. R. Short.

Dr. G. Parker, Mr. A. R. Short and Dr. J. O. Symes were elected to serve on the University Medical Library Sub-Committee, on the proposal of Dr. Gee, seconded by Mr. C. A. Moore.

The President then referred to the resignation of Dr. W. A. Smith from the office of Hon. Medical Librarian on account of ill-health ; and on the proposal of Mr. A. L. Flemming, seconded by Mr. Jackman, Dr. Smith was elected an honorary member of the Society.

---

The second meeting of the session was held in the Physiological Lecture Theatre of the University on Wednesday, 12th November, 1930, at 8.30 p.m. Professor D. C. Rayner in the Chair.

The minutes of the previous meeting were read and confirmed.

Dr. Bergin and Dr. A. D. Fraser showed respectively X-rays and specimens.

Dr. R. S. Statham gave an address on "**Ectopic Pregnancy.**" This paper, with a summary of the ensuing discussion, will appear in our next issue.

The question of admitting non-medical guests to the Society's dinner was raised. It was decided by vote that non-medical guests could not be invited.

---

The third meeting of the session was held in the Physiological Lecture Theatre of the University, on Wednesday, 10th December, 1930, at 8.30 p.m., Professor D. C. Rayner in the Chair.

Cases and X-rays were shown by Drs. D. A. Alexander and G. B. Bush.

Dr. Carey Coombs showed (*a*) a series of sections illustrating the auriculo-ventricular bundle from a normal man, (*b*) a similar series from a case of complete heart block, (*c*) diagrams summarizing researches into the pathology of the principal cardiac infections.

Dr. Herbert Rogers read notes on cases of "**Necrosis of the Myocardium,**" with illustrative specimens.

Dr. Bruce Perry read a paper on "**Congenital Malformations of the Heart as seen in Elementary School Children.**"

These papers, with the discussion that followed, will be published in our next issue.

---

### British Medical Association.

(BATH AND BRISTOL BRANCH.)

ON 29th October a clinical meeting was held in the Medical Library of the University. A large number of cases was shown, together with various X-ray and pathological specimens. The meeting was attended by over a hundred members and visitors.

